# Germinal Centre B Cell Functions and Lymphomagenesis: Circuits Involving MYC and MicroRNAs

**DOI:** 10.3390/cells8111365

**Published:** 2019-10-31

**Authors:** Marcela Cristina Robaina, Luciano Mazzoccoli, Claudete Esteves Klumb

**Affiliations:** Programa de Pesquisa em Hemato-Oncologia Molecular, Coordenação de Pesquisa, Instituto Nacional de Câncer, Rio de Janeiro, CEP: 20230-130, Brazil; mrobaina@ymail.com (M.C.R.); lucianomazzoccoli@gmail.com (L.M.)

**Keywords:** MYC, microRNA, germinal centre-derived lymphomas, feed-forward loop, feedback loop

## Abstract

Background: The transcription factor MYC regulates several biological cellular processes, and its target gene network comprises approximately 15% of all human genes, including microRNAs (miRNAs), that also contribute to MYC regulatory activity. Although miRNAs are emerging as key regulators of immune functions, the specific roles of miRNAs in the regulation/dysregulation of germinal centre B-cells and B-cell lymphomas are still being uncovered. The regulatory network that integrates MYC, target genes and miRNAs is a field of intense study, highlighting potential pathways to be explored in the context of future clinical approaches. Methods: The scientific literature that is indexed in PUBMED was consulted for publications involving MYC and miRNAs with validated bioinformatics analyses or experimental protocols. Additionally, seminal studies on germinal centre B-cell functions and lymphomagenesis were reported. Conclusions: This review summarizes the interactions between MYC and miRNAs through regulatory loops and circuits involving target genes in germinal centre B-cell lymphomas with MYC alterations. Moreover, we provide an overview of the understanding of the regulatory networks between MYC and miRNAs, highlighting the potential implication of this approach for the comprehension of germinal centre B-cell lymphoma pathogenesis. Therefore, circuits involving MYC, target genes and miRNAs provide novel insight into lymphomagenesis that could be useful for new improved therapeutic strategies.

## 1. Introduction

The proto-oncogene c-MYC (MYC) codifies a transcription factor (TF), namely, MYC, that regulates several seminal biological cellular processes. MYC protein expression is tightly regulated, and MYC is only expressed during certain phases of the cell cycle. As a potent TF, MYC, together with its partner protein MAX, induces or represses the expression of hundreds of genes that mediate its biological functions. In fact, the MYC target gene network comprises approximately 15% of all human genes [1,2,3]. MYC deregulation occurs in a wide variety of cancer types through different mechanisms, such as chromosomal translocations [4,5,6], gene amplifications [7], mutations in MYC itself [8], mutations in its upstream regulators [9], and degradation of ubiquitin ligase-mediators, resulting in increased MYC protein stability [10,11]. In addition to regulating the transcription of mRNAs, MYC also regulates the expression of microRNAs (miRNAs) by binding to specific DNA elements in the vicinity of their promoters [12].

MiRNAs are small non-coding RNAs (20–23 nucleotides in length), which bind to the 3′-UTRs of target mRNAs and regulate posttranscriptional expression by either triggering translational repression or the direct cleavage of mRNAs [13,14,15,16,17]. Additionally, MYC signalling pathways involving miRNAs have been associated with several relevant biological processes, such as proliferation, apoptosis, cell cycle progression, angiogenesis, and metabolic reprogramming [18].

A previous study explored MYC-miRNA binding using experimental MYC-DNA binding sites of miRNAs by scanning the putative promoter regions identified by ChIP-PET. The majority of MYC-miRNAs involved in MYC-related cancer pathways were expressed abnormally dysregulating the cell cycle, apoptosis process and cell proliferation [19]. Moreover, numerous feed-forward loops (FFLs) [20] comprising MYC, miRNAs and target genes were identified [19]. The existence of these FFLs indicates a complex MYC-regulatory network where MYC, as a TF, binds to the promoters of target genes via the canonical E-box sequence CACGTG, as well as to non-canonical DNA sequences, including CATGTG [21]. On the other hand, when MYC represses transcription, it recruits Miz-1 or other proteins to bind core promoters [22,23]. Additionally, MYC-regulated miRNAs may also regulate the same target gene regulated by MYC, or the miRNA may suppress the translation of both MYC and its target [24]. The understanding of the most important MYC–miRNA target during lymphomagenesis is beyond the analysis of one pathway, few players, or simple interactions and demands investigations into more complex networks in cellular interactions [25]. Although MYC directly regulates the expression of miRNAs, such as the miR-17–92 cluster, miR-29 family members, miR-34a, miR-26, miR-15a/16-1 and miR-9, the expression of MYC is also negatively regulated by miRNAs, such as miR-34 and let-7a [26,27,28,29]. Alternatively, miRNAs indirectly regulate MYC at the transcriptional and posttranscriptional levels by targeting other MYC-regulatory proteins or miRNAs [30]. Moreover, MYC also induces a number of miRNAs that limit the amplification of their targets [31]. The combination of several networks indicates that the MYC-miRNA regulatory network may be formed by a number of FFLs. Here, based on the involvement of miRNAs in the regulation of B-cell development/function and in lymphomagenesis, we review the roles of miRNAs in normal germinal centre (GC) reactions and focus on relevant FFLs in the context of MYC-regulatory networks in GC-derived B-cell lymphomas.

## 2. Transcriptional Networks Involved in Physiological B-Cell Differentiation

**Figure 1:**
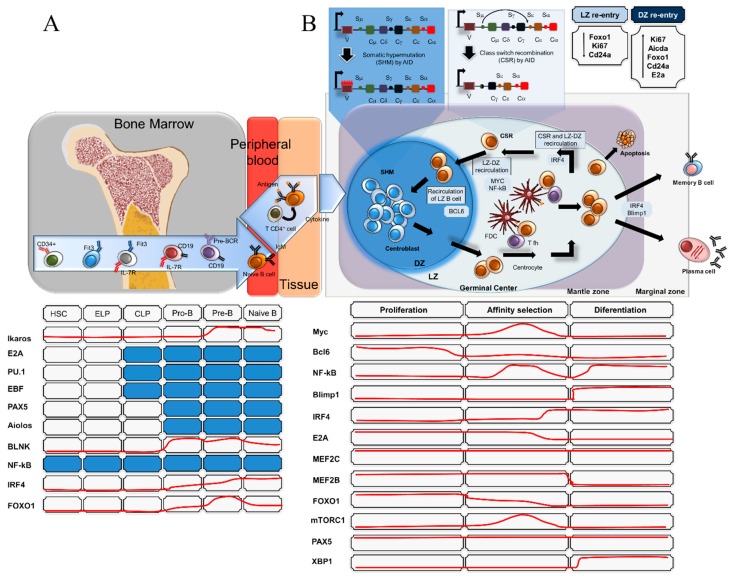
Transcriptional regulators involved in B-cell development and B-cell activation. (**A**) Schematic representation of the transcription factors involved in the process of B-cell development from the bone marrow to the peripheral blood and secondary lymph nodes. Naïve B-cells with successful V(D)J recombination and that express functional B-cell receptors leave the bone marrow and migrate to peripheral lymphoid tissues. (**B**) Once activated by foreign antigens, the B-cells undergo several biological processes, including activation, clonal expansion and somatic hypermutations (SHM) in the dark zone (DZ) of the germinal centres of the lymph nodes, and enter the light zone (LZ) with transcriptional modifications. In the LZ, the follicular T helper (Tfh) contributes to the process of class-switch recombination (CSR) through antigen presentation by the follicular dendritic cells (FDC), re-entering the DZ of the germinal centre. The final process of B-cell activation may lead to death by apoptosis, memory B-cell differentiation or antibody-secreting B-cells (plasma cells). The red lines represent fluctuations in transcriptional factor expression during B-cell development and activation. The blue boxes represent the detected expression in the respective phase of B-cell development.

In mammals, B-cell lineage commitment is initiated from haematopoietic stem cells (HSCs) in the bone marrow. B-cell development is a tightly regulated process in which multiple transcription factors, modulators and extrinsic signals are involved in a set of well-ordered rearrangements at immunoglobulin (Ig) loci. Early B-cell development ends when a B-cell precursor successfully rearranges Ig heavy- and light-chain genes. These rearrangements are generated by sequential V(D)J recombination events in immunoglobulin heavy chain (IgH) and light chain (IgL) genes and occur at distinct stages of precursor B-cell development, a process of differentiation that involves B-cell antigen receptor (BCR) formation (Figure 1A). This subject will not be addressed here because it is beyond the scope of this review, but a comprehensive overview is presented in other reviews [32,33,34,35,36]. Briefly, the maturation of B-cells occurs in the bone marrow in the absence of antigen. The B-cells that have not been exposed to a specific antigen are called naïve B-cells. Mature B-cells with successful V(D)J recombination and that express functional B-cell receptors leave the bone marrow and migrate to peripheral lymphoid tissues. Upon exposure to an antigen in the secondary lymphoid organs, B-cells enter the primary follicle to form the GCs that comprise two different regions that are termed the dark zone (DZ) and light zone (LZ). The DZ predominantly consists of proliferating GC B-cells, whereas the LZ is more sparsely populated by B-cells than the DZ. The DZ and LZ are surrounded by a mantle zone (MZ) which consists of naïve B-cells which were not activated by their antigen and are displaced to the outside of the follicle, where they form an MZ around the GC [37]. When B-cells enter into the DZ of the GC they undergo proliferation and somatic hypermutation (SHM) of their immunoglobulin genes. SHM is conducted by an enzyme named activation-induced deaminase (AID) [38] and is mediated by the encounter of B-cells with antigen-presenting cells and CD4+ T-cells. Through an SHM mechanism, point mutations are introduced in the variable region of the heavy and the light chain. The AID enzyme binds to single-stranded DNA and deaminates cytosine to uracil, activating mismatch repair or base excision. Thus, the process increases the affinity of the B cell receptor (BCR) for the antigen [38]. The transcriptional factor B-cell lymphoma 6 (BCL6), the “master regulator” of GC reaction functions, is a potent transcriptional repressor that regulates GC formation by silencing anti-apoptotic BCL2 during the SHM process (Figure 1B). In addition, after proliferative cycles, DZ B-cells transit to the GC light zone (LZ) and are activated and selected through class-switch recombination (CSR) events, which are also mediated by AID [39]. By CSR, a deletional recombination event, the constant gene segment of the heavy chain locus is switched irreversibly. Following multiple rounds of CSR in the LZ and subsequent re-entry into the DZ, B-cells that express high-affinity antibodies are generated and differentiate into either antibody-secreting plasma cell or memory B-cells [40,41,42]. This stepwise process is regulated by several TFs, as depicted (Figure 1B). The TF BCL6 regulates a bulky transcriptional network that involves multiple signalling pathways, such as the DNA damage response, cell cycle arrest, apoptosis, SHM, CSR, and B-cell activation [43]. On the other hand, even though BCL6 is a transcriptional repressor, it can indirectly promote gene expression by negatively modulating miRNAs [44].

The involvement of MYC in GC B-cell development is also relevant for understanding the role of MYC in GC-derived B-cell lymphomas. Remarkably, the intense proliferation of DZ-GC B-cells in response to T cell-dependent antigens occurs in the absence of MYC expression [45]. MYC suppression occurs via transcriptional repression by BCL6, whereby BCL6 binds to the MYC promoter, resulting in the repression of MYC in DZ B-cells [46,47,48]. It is noteworthy that the expression of MYC in GC B-cells is heavily regulated and restricted to specific cells of GC development. The expression of MYC in GC B-cells is low [45], and paradoxically low levels of MYC mRNA and protein from most GC B-cells seems to be incompatible with the proliferative activity induced by MYC in B-cells. However, it was recently demonstrated that MYC biological activity is required for the initiation and maintenance of the GC reaction [48]. The deletion of MYC in GC B-cells at an early stage of the GC reaction results in complete ablation of GCs in response to T cell-dependent immunization and in impairment of GC formation [49]. Notably, MYC expression is restricted to a subset of LZ B-cells with MYC RNA levels detected to be several-fold higher than those in naïve B-cells. The MYC-positive B-cells in the GC LZ correspond to cells that are being actively selected for their high-affinity BCRs [48]. Thus, mature GC LZ cells are primed for re-entry into the DZ for new rounds of cycle division and maintenance of the GC reaction [48]. One of the TFs regulating the re-entry of these cells into the DZ is MYC. MYC, being repressed by BCL6 after the GC initiation, becomes reactivated in a subset of LZ B-cells, allowing B-cells to undergo further rounds of clonal expansion in the DZ. The final events of the GC reaction and B-cell activation may lead to death by apoptosis, memory B-cell differentiation or antibody-secreting B-cells (plasma cells) [50] (Figure 1B).

## 3. MiRNAs in Germinal Centre B-Cell Functions and Lymphomagenesis

During B-cell differentiation, miRNAs regulate all stages of mammalian B-cell development and activation [51,52]. The ablation of Dicer, the enzyme involved in miRNA biogenesis, results in a block of B-cell development at the pro-B stage in mice [53]. In addition, the analysis of B lymphocytes lacking components of the microprocessor complex (Drosha or DGCR8) also showed a block at the pro- to pre-B cell transition due to increased apoptosis and a failure of pre-B cells to proliferate [54].

Over the last few decades, the roles of miRNAs in B-lymphocyte development have been identified through the miRNA profiling of B-cell subsets and the human mature B-cell miRNome [55,56,57]. Using a combination of cloning and computational analysis, Basso et al. demonstrated that normal B-cell subpopulations as well as malignant B-cells are characterized by specific miRNA “signatures”, suggesting key regulatory roles for miRNAs in B-cell differentiation and transformation [56]. In addition, the differences in the miRNA expression profiles of GC and non-GC B-cells [56] reflect the differences in the coding gene profiles observed by Klein et al. [45].

Another study by Tan et al. revealed that the expression levels of several miRNAs were elevated in GC B-cells compared to naïve and memory B-cells. Alterations of more than 4.5-fold in the expression levels of miR-17-5p, miR-106a, miR-146a, miR-150 and miR-181b were found in GC B-cells, although gradual decreases in the staining intensities of miR-17-5p, miR-106a and miR-181b were observed by miRNA in situ hybridization in GC cells from the DZ to the LZ [55]. Notably, the same study revealed that miR-150 presented more than 10-fold lower expression levels in GC B-cells than in naïve and memory B-cells. This miRNA shows a dynamic expression profile during lymphocyte development and is expressed in all lymphoid tissues, including lymph nodes, the spleen and the thymus. High expression is detected in mature murine B and T-cells but not in their progenitors or upon their activation [58]. Likewise, the downregulation of miR-150 is required for GC selection and for development of the humoural immune response during late B-cell follicular maturation [55,58].

Recently, Schneider et al. investigated the miRNA expression profiles of mature B-cells and GC-derived lymphomas using small RNA sequencing of GC B-cells isolated from human tonsils and primary diffuse large B-cell lymphoma (DLBCL) biopsies [59]. In this study, it was showed that the miR-28 was significantly down-regulated in BL, as well as in other GC-derived B-cell non-Hodgkin lymphomas (NHLs). The reexpression of miR-28 in Burkitt lymphoma (BL) cell lines led to retarded proliferation due to a combination of apoptosis and cell cycle arrest. Besides, MYC silencing in seven BL cell lines using shRNA enhanced the miR-28 expression suggesting that MYC is involved in the miR-28 regulation. In this context, the expression of miR-28 in normal GC B-cells is consistent with the absence of MYC in the same cells as previously mentioned whereas the transformed GC B-cells with high levels of MYC are associated with abnormally downregulated miR-28 expression levels. The findings suggested that miR-28 repression by MYC contributes to B-cell lymphomagenesis.

**Figure 2:**
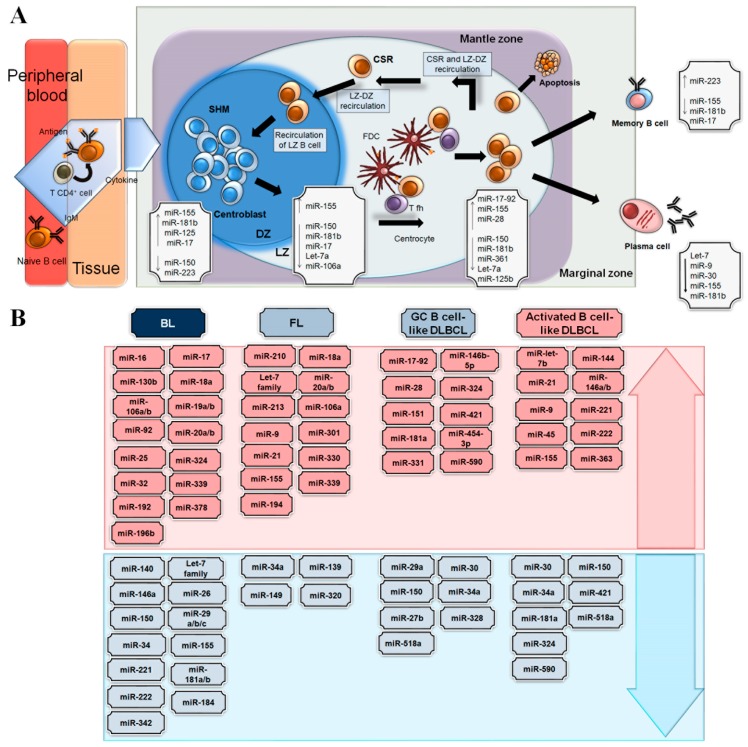
Major miRNAs playing a role in B-cell functions and lymphomagenesis. (**A**) Graphic representation of different miRNAs involved in the B-cell functions in the germinal centre. The insides of the boxes show the miRNAs reported to have relevant roles in the different areas of the germinal centre. (**B**) The miRNAs that are up- (in red) and downregulated (in blue) are described for different B-cell lymphomas: Burkitt lymphoma (BL), follicular lymphoma (FL), germinal centre B cell-like (GCB)-diffuse large B cell lymphoma (DLBL), and activated B cell-like (ABC)-DLBCL.

Regarding miR-17-5p and miR-106a, both originate from the same miRNA family that also comprises miR-17-5p, miR-20a, miR-20b, miR-93, miR-106a and miR-106b and show similar seed sequences to potentially bind to the same target genes [60]. The cyclin-dependent kinase inhibitor CDKN1A, which encodes the p21 protein, has emerged as a target gene that is negatively regulated by miR-17-5p. High expression levels of miR17-5p and miR-106a were detected in the centroblasts of the DZ. On the other hand, the expression of CDKN1A in the centroblasts has been shown to be downregulated 30-fold, suggesting the critical roles of these miRNAs, allowing the progression of DZ-centroblasts from the G1 to S phases of the cell cycle and sustaining proliferation [55]. Notably, GC B-cell formation is extremely impaired in the absence of Dicer as a result of defects in cell proliferation and survival due to elevated levels of pro-apoptotic protein BIM and high transcript levels of several inhibitors of cyclin-dependent kinases (CDKs), with more than 10-fold higher expression of CDKN1C and CDKN2B [61]. The data suggest that Dicer and, probably, miRNAs are critical for GC formation and follicular B-cell development [61]. Among 37 miRNAs that target CDK inhibitors (CDKN1A, CDKN2B, CDKN1C) and that are highly expressed in GC B-cells, some MYC-regulated miRNAs were identified, pointing to fine-tuned miRNA regulation by MYC in specific (LZ) MYC-expressing GC cells, as previously described [48,61] (Figure 2A).

Altogether, for B-cell maturation, the SMH and CSR of immunoglobulin genes are essential mechanisms that depend on miRNA regulation. BIC (pri-miR-155), a primary miRNA, is further processed into mature miR-155, and this miRNA plays a specific role in the control of the GC reaction in the context of a T cell–dependent antibody response. MiR-155 knockout mice have defective B-cell development, characterized by a diminished fraction of GC B-cells and an impaired T cell–dependent antibody response. On the other hand, an increase in GC B-cells was observed in knock-in mice [62,63].

The crucial role of miR-155 in normal B-cell immune physiological functions suggests that its dysregulation may contribute to the pathogenesis of most frequent types of B-cell lymphoma. Kluiver et al. first demonstrated that this miRNA was downregulated in BL [64]. Concurrently, downregulation of miR-155 was also reported in BL tissues (~ 8-fold) compared to other lymphomas [65]. Remarkably, BL cell lines, in contrast to normal B-cells, do not process BIC into mature miR-155 [66]. In addition, miR-155 was found to be 8-fold lower in BL than in other NHLs [65]. MiR-155 suppresses the activation-induced cytidine deaminase gene (AID), a key regulator of SMH in GC. According to the algorithms TargetScan and miRanda, miR-155 is one of the uppermost miRNAs predicted to target AID [67]. AID activity increases the propensity of GC B-cells to undergo lymphomagenic transformation [68,69,70]. Then, downregulation of miR-155 in BL cells may result in an increase in AID expression that promotes *MYC-IGH* translocations, which are the genetic hallmarks of BL [71,72]. Interestingly, although miR-155 has an important role in the control of the GC reaction, paradoxically, it is a negative regulator of AID [71,72]. To resolve this contradiction, Basso et al. demonstrated that BCL6 positively regulates AID gene expression via repression of miR-155 in the DZ, whereas miR-155 expression in the LZ regulates other genes, providing fine spatial-temporal regulation during the GC reaction [44].

In contrast to BL, DLBCL is characterized by overexpression of miR-155 [56,62], suggesting that miR-155 is dependent on the cell type/stage [73]. Likewise, aberrant BCL6 activity may contribute, via miR-155 repression, to sustain the expression of AID that promotes the accumulation of genetic damage and induces BCL6-driven lymphomagenesis [74]. Moreover, miR-181b negatively regulates the CSR reaction by directly targeting AID, downregulating its expression and leading to impaired CSR. Elevated levels of miR-181b impair CSR both in transduced mouse B-cells and in a BL cell line [75] (Figure 2B).

In summary, although miRNAs are emerging as key regulators of immune functions, the specific roles of miRNAs in the regulation/dysregulation of GC B-cells are still being uncovered. Validation studies of miRNAs within particular B-cell subsets or at specific developmental stages are needed to understand the networks involving feedback loops and that are controlled by miRNAs in GC B-cells. In addition, most miRNAs regulate cellular functions through complex mechanisms that involve precise, spatiotemporal gene expression control in association with many other miRNAs and transcription factors [67,76,77]. While miRNAs have important roles in physiological GC B-cell functions, dysregulated miRNAs play key roles in lymphoma development and progression, either as oncogenes (e.g., the miRNA 17-92 cluster members, miR-155, miR-21, and miR-217) or as tumour suppressors (e.g., miR-34a, miR-146, and the miR-29 family) [78,79,80].

In recent years, several studies have reported aberrant expression of miRNAs in B-cell lymphomas due to genetic and epigenetic alterations, such as chromosomal aberrations, epigenetic modifications and mutations in the sequence of miRNAs or their promoter regions, as well as due to factors involved in the miRNA biogenesis machinery that can alter miRNA expression [81]. These studies have shed more light on the dysregulation of miRNA expression in B-cell lymphomas (Figure 2B).

## 4. Germinal Centre-Derived B Cell Lymphomas with Abnormalities Involving MYC

MYC has been identified as a pivotal oncogene in different cancer types, including lymphomas. B-cell lymphomas that have been associated with MYC translocations include BL, DLBCL, follicular lymphoma (FL), plasmablastic lymphoma (PBL) and mantle cell lymphoma (MCL) [82,83].

In the remainder of this review, we will focus only on MYC-abnormality-containing lymphomas that are derived from GC B-cells: BL, DLBCL and FL.

### 4.1. Burkitt Lymphoma

BL, a GC-derived B-cell lymphoma, is the most common subtype of childhood NHL, accounting for 35–40% of cases [84]. There are three subtypes of BL, namely, endemic that occurs in equatorial Africa often in association with endemic malaria, at frequencies of 5–10 cases per million, sporadic occurring worldwide at an approximate frequency of 1–3 cases per million, and immunodeficiency-associated generally diagnosed in patients with HIV infection. The three BL subtypes differ in their association with EBV infection of the tumor cells. However, all subtypes are morphologically indistinguishable [85]. The molecular signature for BL is the detection of the translocated *MYC* gene, which results in the constitutive expression of the MYC protein. The *MYC* gene translocation to the immunoglobulin heavy chain gene locus (14q32) is the most frequent translocation, representing approximately 80% of cases [86]. However, there are two variant translocations that can be found in BL tumours. In these variants, the *MYC* gene is translocated with the immunoglobulin light chain genes at 2p12 or 22q11 [4,5].

**Figure 3:**
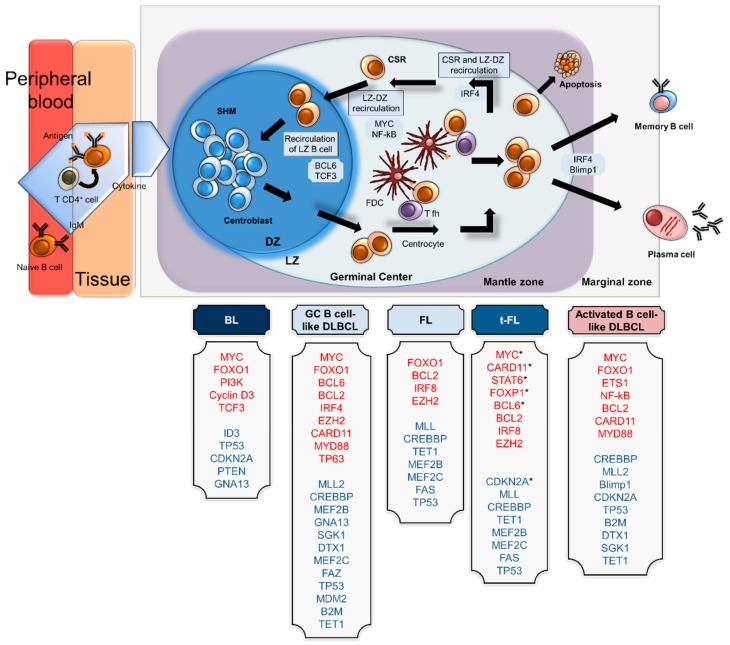
Cellular origin and expression profiles of transcriptional factors in different B-cell lymphomas. Schematic representation of the cellular origins of different subtypes of B-cell lymphomas: Burkitt lymphoma (BL) is reported to originate from centroblasts from the dark zone (DZ); germinal centre B cell-like (GCB)-diffuse large B cell lymphoma (DLBL) and follicular lymphoma (FL) have features resembling cells from the light zone (LZ); and activated B cell-like (ABC)- DLBCL is related to B-cell terminal differentiation before the plasma cell stage. The names of overexpressed genes are indicated in red, and the names of downregulated genes are indicated in blue. * genes reported to be deregulated in t-FL.

The rearrangement of *MYC* is believed to be the first event in BL pathogenesis, and it is often associated with a simple karyotype [87]. *MYC-IG* translocations dysregulate the tightly regulated MYC expression in the GC, leading to constitutive and ectopic expression during the GC reaction. Furthermore, translocated *MYC* is located in the hypermutable immunoglobulin locus and is subject to somatic hypermutations. The mutation usually occurs in exon 1 and contributes to the oncogenic role of MYC by increasing MYC protein stability [88,89]. In recent years, high-resolution genomic analyses have demonstrated that other genetic alterations, such as gain-of-function mutations in *TCF3* and inactivating mutations in *ID3* (the negative regulator of *TCF3*) and in *CNND3*, cooperate with *MYC* in BL pathogenesis [90,91,92] (Figure 3).

### 4.2. Diffuse Large B-Cell Lymphoma

DLBCL is the most common B-cell lymphoma in adults, representing approximately 40% of all lymphomas [85]. DLBCL comprises a heterogeneous group of lymphomas with different phenotypes, biologies and clinical responses. There are two highly frequent subtypes out of 10 distinct large cell lymphomas: one from B-cells arrested in the GC (GCB DLBCL) and another derived from activated B-cells (ABC), the latter being associated with the worst survival outcome [85]. Another subtype can originate from the histological transformation of low-grade lymphomas, such as FL.

The main translocated oncogenes in DLCBL are *BCL6* (B-cell lymphoma 6) and *BCL2* (B-cell CLL/lymphoma 2). *MYC* translocation is detected in approximately 7–14% of DLBCL cases [93] and is associated with a poor outcome [94,95]. DLBCL with a MYC rearrangement has a GCB phenotype (19%) more frequent than ABC (5%) [96]. In contrast to those in BL, *MYC* translocations in DLBCL more commonly encompass the light chain or non-IG partners, followed by a complex karyotype [97,98]. DLBCL with MYC rearrangements or amplification are associated with MYC nuclear expression in more than 70% of cells. MYC protein expression is also detected in a subset of patients (>30-40% positive cells) without gene alterations who have a similar poor prognosis [99]. This observation suggests that mechanisms other than rearrangements, such as miRNA deregulation, could be associated with MYC overexpression in DLBCL [96,100].

Nearly 85% of DLBCL cases show genetic alterations involving genes encoding histone/chromatin modifiers, such as methyltransferase KMT2D (30% of cases), the acetyltransferases CREBBP and EP300 (20% and 5% of cases, respectively), and the polycomb-group oncogene EZH2 (20% of patients) [98] (Figure 3).

### 4.3. Follicular Lymphoma

FL, the second most common lymphoma subtype in adults, is frequently categorized as low-grade NHL and has an indolent course [85]. This lymphoma is characterized by chromosomal translocations involving the proto-oncogene *BCL2* [98]. In the majority of cases, t(14;18) is detectable, and variant translocations t(2;18) and t(18;22) are significantly less frequent but have biological equivalency [101] (Figure 3). In addition to the hallmark *BCL2* translocation, FL is associated with a wide variety of genetic lesions involving chromatin (CREBBP, KMT2D, EZH2) and transcriptional regulators (MEF2B, STAT6, FOXO1) and factors regulating the microenvironment (TNFRSF14) [98] (Figure 3).

Genetic aberrations of *MYC* or its overexpression have been described in FL with histological transformation to a high-grade lymphoma (t-FL) that is associated with a clinically aggressive course and dismal prognosis [102,103,104,105]. t-FL is derived from the GC B-cell-like subtype in the majority of cases (80%) [106]. Approximately 40% of t-FLs show *MYC* mutations/translocations/amplification or gains associated with MYC overexpression and other alterations, such as p53 mutations, deletions, loss of heterozygosity, *CDKN2A* deletions and loss of heterozygosity [105,107,108].

## 5. MYC Feedback Loops Involving microRNAs and Their Roles in B-Cell Lymphomas

The gene regulation network is composed of the association of a small set of network motifs. The motifs comprise partners based on their recurrence in gene regulation, such as miRNAs, transcription factors and target genes, which constitute the basic unit of a network motif [109].

As mentioned previously, the MYC family is composed of members that share a DNA binding ability and promoter transcription. MYC has been described as a noncanonical transcription factor that regulates the transcription of up to 15% of specific target genes involved in several biological processes [110]. Aside from its promoter-dependent regulatory mechanism, MYC also acts in a posttranscriptional manner through miRNA regulation [29]. As described before, miRNAs can weakly bind to the mRNA of a transcribed gene and block its translation, thereby acting as tumour suppressors or oncogenes, depending on the context [111].

**Figure 4:**
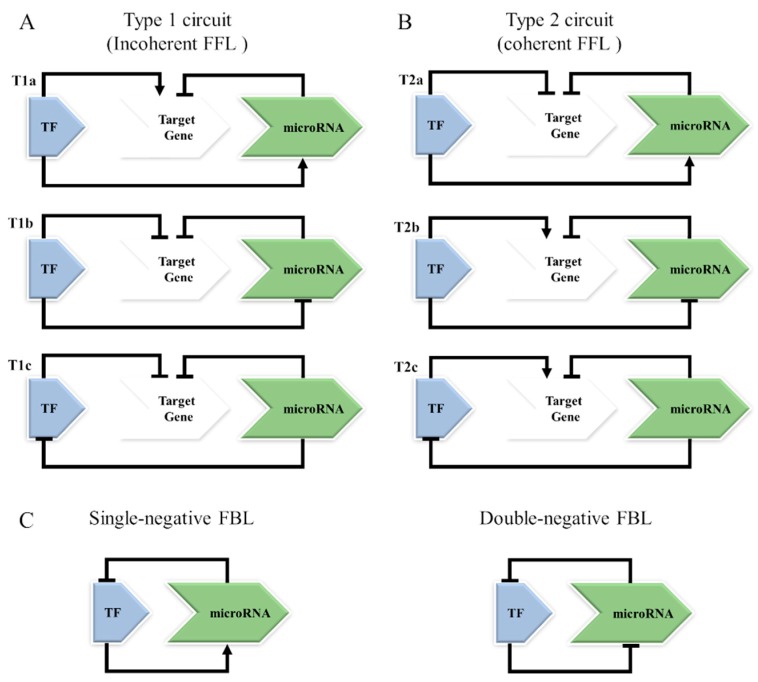
Schematic representation of different networks involving transcriptional factors and miRNAs. (**A**) In type 1 circuits, also known as incoherent feed-forward loops, the TF and miRNA together co-regulate a target gene, but the TF can regulate the miRNA expression or vice versa. The TF can induce miRNA expression (T1a) or repress (T1b) or be repressed by the miRNA (T1c). (**B**) In type 2 circuits, also known as coherent feed-forward loops, the TF and miRNA have the same effects on the target gene, potentially activating or repressing its expression. The TF can repress the target gene and increase the expression of the corresponding miRNA repressor (T2a); the TF induces the target gene and represses its miRNA repressor (T2b); the miRNA that represses a target gene also inhibits its TF (T2c). (**C**) In the feedback loops, the TF and the miRNA regulate different target genes, but one can act by regulating the other (SNF), or both can regulate each other (DNF).

The association of recurrent miRNAs, transcription factors and target genes is the basic unit of a feedback loop (FBL) and the feed-forward loop (FFL) [112,113]. The FFL motif involves the participation of TF regulation of miRNA expression or a miRNA that represses a TF. However, both the TF and miRNA act together to coregulate a target gene. For the FBL motif, the TF and miRNA act by regulating each other, and each of them regulates targets separately [113] (Figure 4).

There are two types of FFLs: incoherent and coherent loops (Figure 4A,B). In the incoherent loop, also called the Type 1 circuit, the expression of a target gene is controlled by two opposing paths, potentially contributing to a fine-tuned regulation of a target gene [114,115]. In the coherent loop, or Type 2 circuit, the components involved have the same effects on the target gene, potentially activating or repressing its expression. Therefore, the biological results from each circuit will be different [115].

In the FBL motif (Figure 4C), the TF and the miRNA regulate each other, and the target gene is regulated separately by them. In a double-negative FBL, the TF and miRNA repress each other, where only one of them is active and the other is repressed, with consequent implications for the cell fate decision [113,116]. In a single-negative FBL, the TF activates the miRNA transcription that, in return, abrogates the translation of the TF mRNA and the increase in its protein levels [117] (Figure 4C).

El Baroudi et al., who analysed independent databases with experimentally validated data, identified several mixed miRNA/transcription factor FFLs regulated by MYC [114]. The analysis was performed with the *Myc Target Gene database* [MYC/Target Gene] [118], *TransmiR* database [MYC/miRNA] [119], *TarBase* [miRNA/Target Gene] [120] and other complementary tools. The study uncovered a total of 143 FFLs involving 29 MYC-regulated miRNAs and 87 joint target genes. Notably, the targets of miRNAs under MYC control were involved in cancer-related pathways [114]. Among these MYC-centred mixed FFLs that were experimentally validated, 15 miRNAs were differentially expressed in BL [65] and were associated with FFLs whose target genes were implicated in relevant cellular pathways, such as proliferation, cycle cell control, apoptosis, metabolism, and methylation (Table 1). These data agree with the study by Igbal et al. that identified 27 miRNA targets of MYC (11 upregulated and 16 downregulated miRNAs), allowing the differentiation of BL (n = 36 patients) from DLBCL (n = 79 patients) [121]. Strikingly, the MYC miRNA effects and targets included the upregulation of miR-17-92 cluster members and their paralogues miR-18b, miR-20a, miR-106 and the downregulation of miRNAs including miR-23a, miR-29c, miR-29b, miR-150 and miR-146a [121], which were experimentally validated [114] and integrate FFLs, as depicted in Table 1. On the other hand, the MYC miRNA targets were significantly associated with BL but not with DLBCL cases with MYC translocations [121], suggesting distinct FFLs involving the MYC–miRNA–target network between BL and DLBCL with MYC translocations. It is important to note that in a previous seminal study by Dave et al., the gene expression profiles of DLBCL cases bearing *MYC* translocations were distinct from those of BL cases [122].

Using murine and human lymphoma models, Bueno et al. investigated the relationship between miRNA expression and the presumed targets of these miRNAs in γ-irradiation-induced mouse lymphomas and in human BL tumours [25]. The analysis of the transcriptional profiles of the γ-irradiation-induced mouse lymphomas identified significant enrichment of upregulated MYC targets. In addition, 41 miRNAs were downregulated in these mouse lymphomas. Since MYC was highly overexpressed in the mouse lymphomas, the downregulated miRNAs included miR-150, miR-22, miR-26a-1, and miR-26b, and the miRNA clusters miR-100–125b-1, miR-99a-125b-2, and miR-29b-2–29c, which are known MYC targets. Remarkably, the *MYC* gene had the highest number of target sites for different downregulated miRNAs, and among them, the let-7 miRNA family members were demonstrated to downregulate MYC expression. The correlation between deregulation of MYC and miRNA levels was also validated in 12 BL tumour samples. Forty-three miRNAs were deregulated in BL samples compared with normal lymph nodes (26 upregulated miRNAs, including the cluster miR-17–92, and 17 miRNAs were downregulated). Moreover, when the interactions between the 17 downregulated miRNAs and the overexpressed genes were mapped, the analysis disclosed MYC as one of the most common targets of the 7 downregulated miRNAs in these tumours. The data were confirmed using luciferase reporter assays by testing 7 miRNAs (including the let-7 family members). Except for miR-155, all these miRNAs were able to bind at the MYC 3′-UTR and downregulate MYC. Thus, the effect of downregulated miRNAs on MYC activity suggests that tumour cells select for the inactivation of several miRNAs that may decrease MYC levels [25]. As previously reported, the main consequence of MYC activation in miRNA biology is widespread miRNA repression [123]. Because some of the silenced miRNAs can also target the MYC transcript, the results suggest regulatory loops between MYC and miRNAs (Figure 4C, double-negative FBL). The silencing of these miRNAs and the consequent enhancement of MYC overexpression results in a lack of balance between MYC and miRNA expression, contributing to the upregulation of multiple MYC targets.

A recent study that investigated miRNA signatures to discriminate B-cell lymphomas that involved known MYC targets showed the miR-17-92 cluster, the miR-29 family, miR-150 and miR-497 to have the highest power of discrimination among the major subtypes. Moreover, MYC-miRNA signatures were associated with MYC protein expression within a range of B-cells and B-cell lymphomas. A network analysis of BL revealed MYC-related miRNAs that were involved in the silencing of the BCL2 protein (miR-17-5p, miR-18a, miR-20a, miR-29 family, let-7 family, miR-34a, miR-34b, miR-125b), a protein whose expression is absent in BL tumours. The BL network also contained CDK6, a validated target of miR-29s, and MYB, CTNNB1, ZEB1, XBP1 and BAX, which remain to be experimentally demonstrated as regulated by MYC-related miRNAs [124]. In this context, it is essential keep in mind the role of circuits in the lymphomagenesis, specifically, in lymphomas with MYC alterations. In sequence, the relevant circuits that were validated and their respectively cellular effects will be addressed in the next section.

## 6. Predicted Models of FFLs among MYC, Its Targets, and miRNAs in Germinal Centre B-Cell Lymphomas

### 6.1. MYC-miR-17, miR-20-E2F1 (Type 1 FFL Circuit)

E2F1 is one of nine members of the E2F family of transcription factors that are known to promote G1-to-S phase progression through the activation of genes involved in cell cycle control and DNA replication. In addition to its role in cell proliferation, E2F1 can induce apoptosis in a p53-dependent and p53-independent manner. E2F1 functions as a growth promoter if pathways that mediate E2F1-induced apoptosis are inactivated [125,126,127,128]. MYC and the E2F1 transcription factors are both implicated in cell cycle progression and linked by common interactor proteins, such as TRRAP and p300/CBP [129,130]. Together, E2F and MYC may activate several genes in a coordinated way, as demonstrated by enrichment for E2F1 binding sites in P493-6 B-cells, a BL model cell line in which MYC is expressed under the control of a tetracycline-regulated promoter [131].

The transcription factor E2F1 is predicted to be regulated by miR-17-5p and miR-20a that arise from the polycistronic transcript c13orf25 located in 13q31.3 [21,132]. MYC binds directly to the miR-17 cluster genomic locus [133,134]. In addition, MYC induces E2F1 gene expression [134,135], and conversely, MYC expression is also induced by E2F1, revealing a putative positive FBL [136]. The negative regulation of E2F1 translation by miR-17-5p and miR-20a provides a mechanism to dampen this mutual activation, promoting strongly controlled expression of MYC and E2F1 gene targets [137]. Thus, MYC simultaneously activates E2F1 transcription and limits its translation, allowing a tightly controlled proliferative signal (Figure 4A T1a).

In sporadic BL tumour samples, as well as in BL cell lines, E2F1 expression is deregulated. The expression levels of E2F1 in BL tumours (n = 30 samples) were found to be 5- to 45-fold higher than those in reactive tonsil samples. With shRNA and E2F1 knockdown strategies, it was demonstrated that the reduction of E2F1 expression inhibits tumour formation, pointing to E2F1 as a key player in BL lymphomagenesis [138].

### 6.2. MYC-miR-29a/b/c-DNMT3B (Type 2 FFL Circuit)

Among a variety of miRNAs with great impact on cancer development, the miR-29 family has been identified as a group of tumour suppressor miRNAs based on their participation in cancer pathways in which they inhibit targets related to proliferation, cell cycle control, apoptosis, angiogenesis, migration, invasion and epigenetic regulation [17,139,140,141].

The miR-29 family has three members (miR-29a, miR-29b and miR-29c) that are transcribed in 2 bi-cistronic clusters: the miR-29a/b-1 cluster is situated on chromosome 7 (7q32), and the miR-29b-2/c cluster is situated on chromosome 1 (1q32). miR-29 family members have similar mature sequences even their genes: miR-29b-1 and miR-29b-2 are located in different parts of the genome, and they have similar mature sequences even though mature miR-29a and miR-29c differ in only a single nucleotide that is outside the seed sequence [142]. An E-box MYC binding site is detected inside the promoter region of both miR-29 clusters. Other TFs, namely, NF-kB and YY1, also negatively regulate miR-29 b/c expression [143,144].

In recent years, miR-29s have been recognized as one of the miRNAs that play a role in BL pathogenesis [145,146,147]. MiR-29s, which are downregulated in BL [65,80,121,148], regulate the expression of miRNA target genes, and their dysregulation leads to an imbalance of cellular pathways [149]. In BL cell line models, MYC binding to miR-29 promoters results in the repression of miRNA expression [123] and altered expression of target genes related to proliferation, cell cycle control, apoptosis, and methylation [146]. In addition, ChIP-PET (ChIP coupled with the paired-end ditag method) identified MYC binding loci in the DNA sequence of DNMT3B followed by transcription activation using a model of human B cell line P493 coupled with gene expression data and computational analysis [131]. As depicted (Figure 4B T2b and Table 1), the type 2 FFL circuit involving MYC – miR-29c – DNMT3B occurs in BL cells [114] (Table 1).

### 6.3. MYC–miR-19a–PTEN (Type 1 or 2 FFL Circuit)

Amplification of the oncogenic miR-17-92 cluster due to 13q31 amplification (C13orf25) occurs more frequently in GCB DLBCL than in ABC-type DLBCL (12.5% versus 0%) [150]. Lenz et al. further showed that DLBCL overexpressing miR-17-92 also expresses MYC and its target genes at significantly higher levels than DLBCL that does not overexpress miR-17-92 [150]. Additionally, Eμ-miR-17~92 transgenic mice develop aggressive DLBCL-like lymphoma [151]. Genomic amplification of 13q32 and/or MYC overexpression can induce upregulation of miR-17-92 cluster members [133,152]. These data suggest that MYC and the miR-17-92 cluster synergistically contribute to lymphoma pathogenesis [152,153]. Thus, although further studies are required to investigate the role of miR-17-92 in DLBCL, a plausible type 2 FFL may involve the proapoptotic *PTEN* tumour suppressor gene, which is a validated miR-19a target [114,154] and a direct MYC target, suggesting that MYC-induced miRNAs may coordinate the balance of cell proliferation and cell death in these lymphomas (Figure 4A T1a). It was shown that the MYC/MAX heterodimer binds directly to the PTEN promoter resulting in PTEN activation [134]. On the other hand, PTEN has been proposed to contribute indirectly to MYC regulation via activation of the PI3K-AKT-GSK3β pathway. Downregulation of the PTEN protein may represent yet an additional pathway of posttranscriptional MYC activation [155].

### 6.4. MYC–miR-150–FOXP1

Regarding FL, a recent study has compared the miRNA profiles of FL and t-FL tumours. The study detected five miRNAs as being differentially expressed, and among them, miR-150 was downregulated in the t-FL samples. Moreover, most t-FL samples showed strong MYC staining, suggesting a relationship between MYC and low levels of miR-150 in the t-FL tumours. The investigation of the mechanisms related to miR-150 downregulation demonstrated that silencing MYC in B-cells by siRNA leads to the upregulation of miR-150 expression in B-cells [156]. ChIP analysis demonstrated the binding of MYC to the region upstream of the miR-150 transcription start site. Another connection was shown where the low levels of miR-150 in t-FL lead to the upregulation of the TF FOXP1 protein, which is a direct target of miR-150 [156]. FOXP1 is a transcriptional activator and repressor of genes involved in the GC reaction. FOXP1 is downregulated in GC B-cells, and aberrant expression of FOXP1 may contribute to B-cell lymphomagenesis [157]. Therefore, MYC-activating genetic aberrations that repress miR-150, which are associated with other alterations such as p53 mutation/deletion, could lead to FOXP1 dysregulation with consequent FL transformation [156,158]. It remains to be elucidated which miRNAs and FFLs contribute to the transformation.

## 7. Remarks and Conclusions

MYC is a TF that regulates several relevant biological cellular events, including miRNA expression. Since each miRNA, in turn, can regulate the expression of hundreds of mRNAs, the circuits involving MYC and miRNAs are complex. It is important to highlight that the robust nature of MYC circuits owes a great deal to miRNA-mediated targeting of multiple genes which makes MYC effects pervasive. The relevance of miRNAs in B-cell functions is a novel insight into lymphomagenesis. Here, we have highlighted the circuits between MYC and miRNAs involved in the pathogenesis of GC-derived B cell lymphomas harboring MYC alterations Additionally, the validation of circuits involving MYC and miRNAs could be useful for improvement of therapeutic strategies.

## Figures and Tables

**Table 1:** The most studied and validated MYC-miRNA circuits in B-cell lymphomas.

miRNA	MYC-miRNA Effect	Target Gene	Cellular Pathways	MYC-Transcription Factor Effect	Type FFL*
hsa-miR-17	Activation	E2F1	regulation of apoptosis and cell cycle	Activation	T1a
	Activation	VEGF	regulation of apoptosis and proliferation	Activation or Repression	T1a or T2a
hsa-miR-19a	Activation	PTEN	regulation of apoptosis, cell cycle and proliferation	Activation or Repression	T1a or T2a
hsa-miR-20a	Activation	E2F1	regulation of apoptosis and cell cycle	Activation	T1a
	Activation	TGFBR2	regulation of cell proliferation	Repression	T2a
	Activation	VEGF	regulation of apoptosis and proliferation	Activation or Repression	T1a or T2a
hsa-miR-106a	Activation	RB1	regulation of apoptosis, cell cycle and proliferation	Repression	T2a
	Activation	VEGF	regulation of apoptosis and proliferation	Activation or Repression	T1a or T2a
	Activation	CDKN1A	regulation of apoptosis, cell cycle and proliferation	Repression	T2a
hsa-miR-106b	Activation	CDKN1A	regulation of apoptosis, cell cycle and proliferation	Repression	T2a
	Activation	E2F1	regulation of apoptosis and cell cycle	Activation	T1a
	Activation	PTEN	regulation of apoptosis, cell cycle and proliferation	Activation or Repression	T1a or T2a
	Activation	VEGF	regulation of apoptosis and proliferation	Activation or Repression	T1a or T2a
hsa-let-7a	Repression	NRAS	regulation of cell proliferation	Activation	T2b
	Repression	CASP3	induction of apoptosis	Repression	T1b
hsa-miR-15a	Repression	VEGF	regulation of apoptosis and proliferation	Activation or Repression	T1b or T2b
hsa-miR-22	Repression	PTEN	regulation of apoptosis, cell cycle and proliferation	Activation or Repression	T1b or T2b
hsa-miR-26a	Repression	PTEN	regulation of apoptosis, cell cycle and proliferation	Activation or Repression	T1b or T2b
hsa-miR-29a	Repression	DNMT3B	regulation of DNA methylation	Activation	T2b
hsa-miR-29b	Repression	DNMT3B	regulation of DNA methylation	Activation	T2b
hsa-miR-29c	Repression	DNMT3B	regulation of DNA methylation	Activation	T2b
hsa-miR-34a	Repression	CCND1	regulation of cell proliferation and cell cycle	Repression	T1b
	Repression	E2F3	regulation of cell proliferation and cell cycle	Activation	T2b
	Repression	MYC	proliferation, cycle cell control, apoptosis, metabolism, and methylation	Repression	T1c

FFL*, feed forward loop type, schematic representations are described in Figure 4.
